# Glutamine metabolism in tumor metastasis: Genes, mechanisms and the therapeutic targets

**DOI:** 10.1016/j.heliyon.2023.e20656

**Published:** 2023-10-05

**Authors:** Xugang Zhong, Zeju He, Li Yin, Yong Fan, Yu Tong, Yao Kang, Qing Bi

**Affiliations:** a Department of Orthopedics, Zhejiang Provincial People's Hospital, Hangzhou, China; b Department of Orthopedics, The Second Affiliated Hospital of Wenzhou Medical University, Wenzhou, China; c Department of Orthopedics, Hangzhou Medical College People's Hospital, Hangzhou, China

**Keywords:** Amino acid metabolism, Glutamine, Cancer metastasis, Metabolic therapy, Precision medicine

## Abstract

Cancer cells frequently change their metabolism from aerobic glycolysis to lipid metabolism and amino acid metabolism to adapt to the malignant biological behaviours of infinite proliferation and distant metastasis. The significance of metabolic substances and patterns in tumour cell metastasis is becoming increasingly prominent. Tumour metastasis involves a series of significant steps such as the shedding of cancer cells from a primary tumour, resistance to apoptosis, and colonisation of metastatic sites. However, the role of glutamine in these processes remains unclear. This review summarises the key enzymes and transporters involved in glutamine metabolism that are related to the pathogenesis of malignant tumour metastasis. We also list the roles of glutamine in resisting oxidative stress and promoting immune escape. Finally, the significance of targeting glutamine metabolism in inhibiting tumour metastasis was proposed, research in this field improving our understanding of amino acid metabolism rewiring and simultaneously bringing about new and exciting therapeutic prospects.

## 1 Introduction

Compared to normal cells, tumour cells exhibit uncontrolled proliferation, increased invasiveness, and distant metastasis [1]. Behind these characteristics are enhanced metabolic intensity and changes in the metabolic mode of tumour cells. One of the more important aspects is the increase in amino acid metabolism [2]. Glutamine is an important amino acid in the human body. In 1935, when Krebs proposed the famous tricarboxylic acid (TCA) cycle [3], people realised its importance as a metabolic substance. Although glutamine is an essential amino acid, the circulating glutamine is the most abundant amino acid in the human body [4]. Given that more than 20 % of the free amino acid pool is in the blood and 40 % is in muscles, a high glutamine concentration provides sufficient carbon and nitrogen sources [5]. When tumour cells specifically convert glucose into lactic acid and secrete it, glutamine supports the mitochondrial oxidative metabolism of tumour cells and is used to maintain their energy metabolism and intracellular homeostasis [6]. In addition, glutamine is conducive to a series of cellular biological activities such as amino acid synthesis [7], cell signal transduction [8], oxidative stress resistance [9], and immune escape of tumour cells [10]. Many studies have focused on the relationship between glutamine and tumour metastasis, showing that glutamine metabolism plays an important role in tumour metastasis.

Distant metastasis, a unique biological feature of malignant tumours, is associated with advanced tumour staging and poor prognosis [11]. Metastasis can lead to failure of tumour treatment and a great increase in mortality, thereby reducing the patients' quality of life. In addition, patients may have corresponding clinical diseases according to different metastatic sites [12]. Bone metastases can lead to pathological fractures, bone pain, hypercalcaemia, and other manifestations. Pulmonary metastasis leads to haemoptysis, dyspnoea, neurovascular compression, intestinal obstruction, indigestion, and malabsorption in gastrointestinal metastasis and becomes cachexia [13], increasing patients' pain and their social and medical burdens. Regarding the occurrence of metastasis, around 1900, James Ewing proposed the theory of circular seeding metastasis [14], after Stephen Paget put forward the famous "seed and soil" hypothesis, pointing out metastatic cancer cells only in particularly suitable tissue "seeding", similar to seeding in "fertile soil" [15]. Glutamine as an important part of the influence of tumour metastasis [16], can promote the occurrence of tumour metastasis through the joint action of multiple aspects. Glutamine metabolism can provide enough energy for the circulating spread of tumour cells, protect tumour cells from anoikis apoptosis, and protect tumour cells from immune attack in a colonisation microenvironment [17]. Competitive glutamine uptake by tumours ensures self-growth and proliferation, leads to low-energy metabolism in immune cells, and eventually results in the loss of the capacity to kill tumour cells [18].

Targeting glutamine metabolism is beneficial for reducing tumour metastasis. This study focused on glutamine-metabolising enzymes and transporters to reveal the effect of glutamine on tumour cell metastasis [19]. At the same time, we focused on the role of glutamine in tumour metastasis and the influence on and mechanism of glutamine metabolism in tumour metastasis through energy metabolism, oxidative stress, and immune evasion, and laid a theoretical foundation for the subsequent targeted glutamine metabolism to reduce tumour metastasis and improve the poor prognosis of tumour patients. We also clarified the limitations of this knowledge and propose possible future directions of development for this field.

## 2 Effect of glutamine on tumour metastasis through synthesis and metabolism

Since the 1950s, it has been noted that tumour cells consume large amounts of glutamine [20]. A series of follow-up studies described the role of glutamine in the TCA carbon replenishment pathway, NADPH production, nucleic acid synthesis, and other aspects. The effect of glutamine on tumour metastasis was also discussed. Glutamine metabolism is considered a positive factor in promoting tumour metastasis. Compared with minimally invasive OVCA, highly invasive OVCA relies more on glutamine metabolism to promote tumour metastasis. Glutamine decomposition is associated with distant metastasis and a low survival rate in patients with OVCA [21]. In addition, the amount of lactic acid released gradually increases from non-neoplastic melanoma cells to metastatic melanoma cells, most of which originates from glutamine catabolism. However, the degree of migration of metastatic melanoma cells mostly depends on the activity of glutamine-metabolising enzymes [22]. Therefore, various metabolic enzymes and amino acid transporters play different roles in the effect of glutamine metabolism on tumour cell metastasis.

### 2.1 GLS

Glutaminase (GLS), one of the most important enzymes in glutamine metabolism, is a component of glutamine utilisation that participates in the TCA cycle, producing ATP and NADPH, and maintains glutathione homeostasis and reactive oxygen species (ROS) in cells. Mammals mostly have two isozymes, GLS1 and GLS2. Although most cell lines co-express the transcripts of the two genes, the effects of the two types of GLSs on tumour metastasis are different.

#### 2.1.1 GLS1

Xiang et al. found that high GLS1 expression in colorectal cancer patients was significantly related to lymph node metastasis and advanced clinical stages. After analysing the cancer genome map database, it was concluded that the increase in GLS1 expression is related to the hypoxic characteristics of tumour cells, which increases the risk of metastasis and death in patients with tumours. The expression of GLS1 is necessary for hypoxia-induced migration and invasion *in vitro* and tumour growth and metastasis *in vivo* [23]. In addition, in the study of cancer stem cells in hepatocellular carcinoma (HCC), the up-regulation of GLS1 was positively correlated with the late clinicopathological features and dry phenotype. Moreover, the stem cell phenotype is an important factor for the recurrence and metastasis of liver cancer cells. GLS1 regulates the dryness characteristics through ROS/Wnt/β-catenin signalling, and targeting GLS1 reduces the expression of HCC dryness-related genes and inhibits tumour metastasis in HCC [24].

**Fig. 1:**
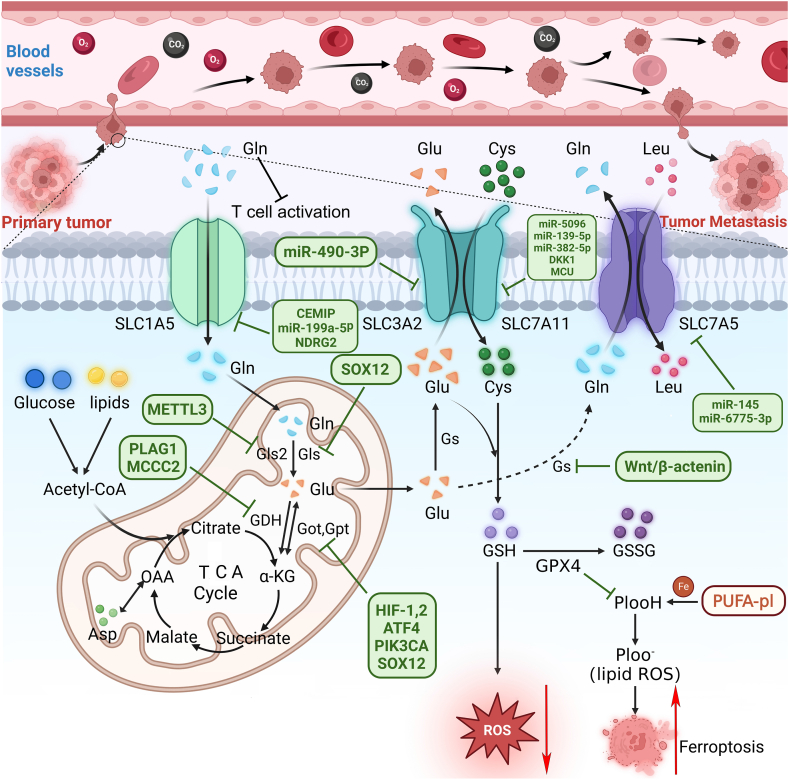
Effects of glutamine metabolising enzymes and transporters on *in situ* tumour metastasis. Tumours undergo metabolic reprogramming to make them more dependent on amino acid metabolism, such as that of glutamine. Glutamine enters the cell body through SLC1A5, is transported to the mitochondria, and is metabolised to glutamic acid by glutaminase. It is the precursor of α-ketoglutarate (α-KG) recycled by tricarboxylic acid (TCA) and is a substrate for glutathione biosynthesis. Glutathione is closely associated with oxidative stress and iron-related death. SLC7A11 and SLC7A5 form intracellular and extracellular amino acid schedules with glutamine, glutamic acid, and other amino acids. High glutamine concentrations in the microenvironment can activate immune cells. The green box indicates the genes that interact with glutamine-metabolising enzymes or transporters. (For interpretation of the references to color in this figure legend, the reader is referred to the Web version of this article.)

In addition to GLS1 itself promoting tumour cell metastasis, GLS1 acts as an intermediate molecule to promote tumour cell metastasis. Clinical statistical analysis shows that the overexpression of SOX12 promotes the proliferation and metastasis of colorectal cancer (CRC) cells by activating a variety of glutamine-metabolising enzymes, including GLS1, mitochondrial aspartate aminotransferase (GOT2), and ASNS (Fig. 1) [25]. Finally, researchers have found that antisense LNC RNA-GLS-AS, which is negatively correlated with GLS1 expression in clinical pancreatic cancer samples, can form double-stranded RNA with GLS1 pre-mRNA through ADAR/Dicer-dependent RNA interference and inhibit GLS1 expression at the post-transcriptional level. Eventually, the proliferation and invasion of pancreatic cancer cells are inhibited by inhibiting the Myc/GLS1 pathway [26].

#### 2.1.2 GLS2

Compared to GLS1, GLS2 is more diverse in promoting or inhibiting tumour metastasis. Zhang et al. found that GLS2 reduces the invasiveness of HCC cells in many ways. First, GLS2 can competitively bind to the small GTP enzyme Rac1 with guanine nucleotide exchange factor, an agonist of Rac1, and then inactivate Rac1, thus promoting P53-mediated tumour metastasis inhibition [27]. Second, GLS2 can promote the maturation of miR-34a by binding and stabilising Dicer and inhibiting the epithelial-mesenchymal transition (EMT) molecule Snail in an activity-dependent manner via miR-34a and glutamine, thus inhibiting tumour metastasis [28].

However, Ramirez-Peña et al. observed through the TCGA and ICGC databases that higher GLS2 expression in breast cancer indicated increased tumour metastasis. The experimental data verified this hypothesis. Compared with the control group, the experimental group injected with tumour cells that overexpressed GLS2 *in situ* showed more lung metastatic nodules when the tumour size *in situ* was the same; this is related to GLS2 promoting ERK/EGF/ZEB1 axis, which finally leads to an increase in EMT-related molecule-vimentin expression and promotes tumour metastasis [29]. In oesophageal squamous cell carcinoma, GLS2, as an intermediate molecule, is regulated by the methylation of METTL3, which eventually promotes tumour cell metastasis (Fig. 1). The METTL3/GLS2 signalling pathway has been identified as a potential target for anti-oesophageal squamous cell carcinoma (ESCC) metastasis [30].

### 2.2 SLC1A5

With the rise and deepening of glutamine metabolism research, SLC1A5 (ASCT2) has gradually become known as the main glutamine transporter, and increasing evidence has shown that SLC1A5 plays important roles in tumour metastasis. These include mediating ATP production and glutathione synthesis induced by glutamine, alleviating tumour cell growth under nutritional pressure, and endowing tumour cells with chemotherapy drug resistance [31]. In a comparative clinical trial of primary versus lymph node metastatic tumours, Csanadi et al. found a positive correlation between SLC1A5 expression and corresponding lymph node metastasis. In addition, SLC1A5 overexpression was found to be a separate prognostic factor when assessed only for lymph node metastasis. SLC1A5 overexpression is associated with a shorter overall survival [32].

In addition, Wang et al. reported that after the prostate cancer cells of shASCT2 were subcutaneously injected into nude mice, under the control of other variables, the tumour size *in situ* in the nude mice was significantly reduced, and lung and liver metastases were significantly reduced [33]. In addition, in CRC, researchers found a new protein named metastasis-related protein, cell migration-inducing and hyaluronan-binding protein (CEMIP), which can regulate the expression of SLC1A5 and SLC38A2 by inhibiting the wnt/β-catenin signal axis (Fig. 1) and thus reduce tumour ATP synthesis by reducing glutamine uptake and metabolism, resulting in a decrease in CRC tumour metastasis [34]. In a study on the inhibition of lung cancer metastasis by inspiratory hyperoxia (IH) treatment, it was found that IH could reduce the expression of SLC1A5 by inhibiting MYC. The researchers showed through luciferase-binding experiments that MYC binds to the SLC1A5 promoter to initiate SLC1A5 transcription and promote glutamine uptake, to finally achieve tumour metastasis [35]. Zhuang et al. found that ABHD11-AS1 (lncRNA-ABHD11 antisense RNA 1), a competitive endogenous RNA, enhanced cell proliferation, migration, and invasion in papillary thyroid cancer (PTC) by regulating the MiR-199A-5p/SLC1A 5 axis. However, previous studies have shown that miR-199a-5p can significantly inhibit cell migration and invasion by binding to the 3′UTR of SNAI1 in PTC (Fig. 1). Interestingly, these phenomena were suppressed by silencing SLC1A5. This proved that the promotion and inhibition of tumour metastasis by ABHD11-AS1 and miR-199a-5p were achieved through SLC1A5 [36]. Mucoepidermoid carcinoma (MEC) is one of the most common primary malignant salivary gland tumours of the oral and maxillofacial regions.

Recently, a tumour suppressor gene, NDRG2, has been identified that belongs to the downstream gene family of N-Myc [37]. Tumour metastasis can be inhibited by SLC1A5 inhibition. The specific mechanism may be that NDRG2 inhibits c-Myc by inducing Fbw7, an E3 ubiquitin ligase-dependent c-Myc degradation protein. NDRG2 can also inhibit c-Myc expression by inhibiting the activation of Akt and GSK3β, thus jointly achieving the purpose of inhibiting c-Myc-mediated transcriptional regulation of ASCT2 (Fig. 1). This eventually leads to weakening of metastasis. Overexpression of SLC1A5 restores the attenuation of tumour metastasis by NDRG2 [38].

### 2.3 SLC7A5

SLC7A5, a glutamine reverse transporter, plays a role in the reverse transport of intracellular glutamine and extracellular leucine. In previous studies, SLC7A5 has been used as a marker for the independent prognosis of multiple tumours. High expression was observed in metastatic samples from more than ten cancers, including metastatic gastric cancer, endometrial-like cancer, advanced laryngeal squamous cell carcinoma, pulmonary neuroendocrine tumour, tongue cancer, colorectal cancer, cholangiocarcinoma, human epithelial ovarian cancer, non-small cell lung cancer, and human clear cell renal cell carcinoma [[39], [40], [41], [42], [43], [44], [45], [46], [47], [48], [49]]. In addition, the anticancer non-coding RNA miR-145, whose overexpression results in the downregulation of various genes, including SLC7A5, significantly inhibits the migration and invasion of glioma cells (Fig. 1) [50].

In gastric cancer, a new oncogene, GSE1, can positively regulate the expression of SLC7A5 in human gastric cancer cells by increasing the stability of the SLC7A5 transcript through a post-transcriptional regulatory mechanism and promoting tumour cell proliferation and metastasis, which was lost after knockdown by shSLC7A5 [51]. In a study on miRNAs regulating tumour progression, miR-6775-3p was found to play a tumour-inhibitory role in ESCC, including the inhibition of growth and liver metastasis of ESCC xenografts. A good clinical prognosis was achieved when miR-6775-3p is highly expressed in patients (Fig. 1). MiR-6775-3p inhibits the expression of the tumour antigen MAGE-A family by directly binding to the 3′UTR region of MAGE-A mRNAs, and attenuates the effect of MAGE-A in inhibiting the transcriptional activity of the tumour suppressor gene *TP53*. In addition, miR-6775-3p can inhibit SLC7A5 expression.

Interestingly, inhibition of miR-6775-3p and SLC7A5 can directly induce p53 transcription. The interaction between the two forms of positive feedback further inhibits the tumour and delays metastasis progression [52]. Finally, in a comparative study involving multiple primary and metastatic cancers, SLC7A5 and CD98 were the most differentially expressed at primary and metastatic sites by immunohistochemistry (IHC). SLC7A5 was significantly associated with distant angiogenesis in enrichment analysis [53].

### 2.4 GDH

Glutamate dehydrogenase (GDH), an important ring in the glutamine metabolic pathway, reversibly oxidises and deaminates glutamic acid to α-ketoglutaric acid (α-KG), which serves as the key catalytic enzyme for glutamine to enter the TCA cycle. While developing lung cancer-targeted drugs, Liu et al. found that GDH was upregulated by pleomorphic adenoma gene 1 (PLAG1). Its product α-KG, activated CaMKK2 by enhancing its indirect binding with AMPK. Simultaneously, CaMKK2 activated downstream AMPK, finally providing anti-anoikis and pro-metastatic signals in LKB1-deficient lung cancer, resulting in a poor prognostic outcome for LKB1-deficient lung cancer patient (Fig. 1). Interestingly, this effect was not related to ROS levels in the cells, and antioxidants alone did not alter the lost resistance to anoikis apoptosis due to GDH knockdown. However, rapamycin, an mTOR inhibitor, resulted in the reversal of enhanced anoikis and reduced ATP levels in GDH-knockout cells, suggesting that GDH may promote tumour metastasis through the activation of AMPK by CamKK2 and subsequent suppression of mTOR signalling [54]. A clinical study on CRC found that GDH expression can be considered an independent prognostic factor for CRC. GDH promotes STAT3-mediated EMT induction and the proliferation, migration, and invasion of CRC cells *in vitro* and *in vivo* [55].

Furthermore, it was found that compared with the simple expression of a certain genotype, high expression of GDH can also be combined with other factors to more effectively indicate poor prognostic outcomes, such as tumour metastasis. For example, there was no significant correlation between individual expression of GDH and SLC25A13, but higher expression of GDH in combination with lower expression of SLC25A13 was more strongly associated with tumour invasion than individual expression When glucose depletion [56]. In a study on prognostic markers of extrahepatic cholangiocarcinoma, the expression level of GDH was found to be related to the malignant progression of various tumours, including lymph node metastasis. It was confirmed that GDH promoted cell proliferation and metastasis through the Smad-mediated TGF-β signalling pathway, providing a new target for new treatment of extrahepatic cholangiocarcinoma [57]. In addition, in a study on the different metabolic characteristics of breast cancer due to different metastatic sites, glutamine metabolic enzymes, including GDH, were significantly increased in breast cancer bone and liver metastases, whereas the glycolytic phenotype was mainly increased in brain and lung metastases. The effects of glutamine metabolism on tumour metastasis and metastatic sites were further clarified [58]. In a study on the effect and mechanism of methyl crotonyl-CoA carboxylase 2 (MCCC2) in prostate cancer, Wang et al. found that MCCC2 is in direct proportion to GDH expression and promotes the proliferation, migration, and invasion of prostate cancer cells by regulating the GDH-p38 -MAPK signaling pathway (Fig. 1). This leads to adverse outcomes such as tumour metastasis [59].

In a study on drug resistance and EMT-mediated tumour metastasis in non-small cell lung cancer, Wang et al. selected GDH as a common contributing factor. It is believed that NSCLC increases oxidative phosphorylation by overexpressing GDH, leading to high resistance, and triggering migration and invasion by inducing Snail. The *in vitro* GDH inhibitor R162 has also been used to overcome drug resistance and extensive *in vivo* metastasis. In addition to clarifying the mechanism, it also provides potential treatment strategies [60]. However, a different outcome was observed in clear cell renal cell carcinoma (ccRCC), with investigators performing GEO/TCGA/UALCAN database analysis and IHC and WB validation, showing that GDH levels were downregulated in ccRCC tissues. Overexpression of GDH in ccRCC was found to inhibit the proliferation and metastasis of RCC cells by inhibiting activation of the PI3K/Akt/mTOR pathway. In addition, GLUD1 level is negatively associated with immunosuppressive microenvironment in ccRCC. These results suggest a negative correlation between GDH and adverse outcomes such as metastasis in ccRCC [61].

### 2.5 GOT GPT

Glutamate-oxaloacetate transaminase (GOT) and glutamate pyruvate transaminase (GPT) are the most important transaminases in the body. Their function has been fully elucidated in a series of tumours and non-tumours. As the most common biochemical indicators in clinical practice, they have multiple biological functions such as indicating liver function, releasing the degree of inflammatory factors, evaluating drug toxicity, and revealing tumour prognosis. However, their function is yet to be fully described and clarified in aspects such as tumour metastasis.

In oral squamous cell carcinoma (OSCC), Li et al. compared extracellular vesicles derived from human primary tumour cells and matched lymph node metastatic (LN1) cells and a related database, and identified seven factors, including GOT1, in 670 kinds of proteins, 217 types of miRNAs, 26 kinds of metabolites, and 63 kinds of lipids, associated with decreased survival rate and increased tumour invasiveness in patients with cancer [62]. In another study of human ductal adenocarcinoma of the pancreas, hypoxia-inducible factor-2a (HIF-2a) was identified as a factor leading to metastases and poor clinical outcomes in human ductal adenocarcinoma of the pancreas (Fig. 1). It promotes the expression of GOT1 by activating the PI3K/mTORC2 pathway. This results in increased glutamic acid production and eventually enhances the atypical metabolism of glutamine [63].

However, in a study on the inhibition of cancer progression, researchers confirmed that α-ketoglutarate esters elicit death of OXPHOS-deficient cancer cells by elevating intracellular aKG and thereby sequestering nitrogen from aspartate through GOT1, leading to the interruption of oxidative phosphorylation progression in *in situ* and metastatic cancer cells and their death [64]. In addition to affecting glutamine metabolism, GOT2 promotes the metabolism of other amino acids and tumour metastasis. For example, SOX12, a tumour metastasis-promoting factor, promotes the formation of aspartic acid and eventually leads to tumour metastasis by upregulating various glutamine metabolic enzymes, including GOT2. The knockout of GOT2 weakens SOX12-induced tumour metastasis (Fig. 1). GOT2 overexpression weakens the inhibition of tumour metastasis by SOX12 knockdown [65]. Interestingly, in contrast to the results described above, low GOT2 expression was associated with tumour metastasis and poor prognosis in a study on HCC using 48 mouse HCC models. GOT2 knockout promotes haematogenous and hepatic tumour metastases. Low GOT2 expression increases glutamine utilisation, nucleotide formation, and GSH synthesis. Therefore, cancer cells have sufficient energy and are protected from oxidative stress death as well as the activation of the classical PI3K/AKT/mTOR pathway to promote the progression of HCC. Finally, low GOT2 expression resensitizes tumour cells to glutaminase inhibitors, to increase the effect of targeted glutamine on tumours [66,67].

#### 2.5.1 Compared with GOT, GPT has a more consistent role in tumour metastasis

In a study on the metastatic potential of lung cancer, researchers isolated a subpopulation of cells labeled with CD133 from A549 cell lines by flow cytometry and demonstrated that CD133^+^ cells had a higher potential for liver metastasis. Simultaneously, they found that the cell line upregulated various metabolic enzymes, including GPT1. A relationship between GPT1 upregulation and metastasis to specific tumour sites was reported [67]. In another study, Mitra et al. found that a muramyl dipeptide derivative (B30-MDP) eliminated tumour immune resistance in highly metastatic L5178Y-ML25 mouse lymphoma cells. When GPT1 and GOT1 levels were decreased in mice, fewer tumour metastases were formed by tumour cell injection than in controls. This indicated the effect of glutamine transaminase levels on primary tumour metastasis [68].

Regarding the specific signalling pathway, control cells showed increased phosphorylation of the mTORC1 substrate p70s6K and hyperphosphorylated 4EBP1 protein enrichment compared to GPT2 knockout cells in a study involving triple-negative breast cancer. GPT2 knockdown can lead to impaired activity in tumour cell mTORC1, which promotes autophagy and reduces the proliferation and metastasis of tumour cells [69]. In another study, researchers found that glutamine deprivation or GLS inhibition upregulated the production of ATF4 and eventually induced GPT2 expression by increasing ROS production (Fig. 1). Increased GPT2 expression can maintain the 10.13039/100004915TCA cycle catch-up to support cell proliferation and metastasis during glutamine deprivation or GLS inhibition. Addition of the antioxidant N-acetylcysteine (NAC) significantly reduced the increase in GPT2 caused by glutamine deprivation or GLS inhibition. Finally, the combined effects of GLS1 and GPT2 in response to compensatory proliferation and distant metastasis of cells during nutritional crisis have been elaborated [70].

Many studies have focused on the interaction between hypoxia-inducible factor (HIF) and GPT2, which leads to tumour metastasis. In a study on HIF responding to hypoxia and promoting tumour cell metastasis by activating the transcription of glutamine metabolic enzyme in tumour cells, it was pointed out that in human glioblastoma (GBM), hypoxia could bind to the hypoxia response element of human GPT2 gene through hypoxia-inducible factor 1 (HIF1) rather than HIF2, leading to increased GPT2 transcription and promoting tumour cell proliferation and metastasis under hypoxia. Genetic or pharmacological inhibition of GPT2 reduces growth and migration of GBM cells. This further explains the effect of GPT2 on tumour growth and metastasis [71]. In another study on breast cancer, Wang et al. found that GPT2 overexpression increased the subpopulations of breast cancer stem cells *in vitro* and promoted tumourigenesis and metastasis in mice *in vivo*. GPT2 inhibits proline hydroxylase 2 (PHD2) activity by regulating HIF1α stability and reducing α-KG levels. The GPT2-α-KG-PHD2 axis leads to the accumulation of HIF1α, constituting the sonic hedgehog signalling pathway. This eventually leads to tumour progression and metastasis [72].

Furthermore, GPT2 cooperates with glutamine transporters to promote tumour metastasis. In a study identifying colorectal signet ring cell carcinoma (SRCC), measurement of the uptake of three nutrients, glucose, fatty acids, and glutamine, by cancer cells revealed the metabolic profile of SRCC. SRCC increase glutamine uptake and metabolism by upregulating GPT2 and SLC1A5, leading to tumour resistance and malignant progression. GPT2 and SLC1A5 inhibitors may inhibit tumour growth and distant metastasis, and increase susceptibility to 5-fu and L-OHP therapy [73].

Finally, multiple studies have found that the *PIK3CA* mutations increase glutamine dependence by upregulating GPT2 and reprogramming glutamine metabolism in CRC cells (Fig. 1). It was also found that *PIK3CA* influences GPT2 through multiple pathways, such as MEK and PDK1. The combination of PI3K-MEK/PDK1-GPT2 inhibitors inhibited CRC cell growth and metastasis more effectively than the inhibitors alone [74].

### 2.6 GS

Glutamine synthetase (GS), a key enzyme in the intracellular synthesis of glutamine, catalyzes glutamine production using ammonium chloride and glutamic acid under an energy supply of ATP. GS expression promotes asparagine and nucleotide synthesis and enhances the uptake of essential amino acids by the extracellular region of the TME [75]. GS can also affect glutamine metabolism by activating, inhibiting, or inhibiting multiple signalling pathways. Finally, tumour cell proliferation, migration, and invasion are altered.

In a trial on the clinical significance of GS, it was concluded from *in vivo* and *in vitro* experiments that GS could promote the metastasis of HCC cells by promoting the expression of EMT molecules [76]. Previous studies have shown that tumour-associated macrophages (TAMs) are involved in proliferation, survival, and metastasis of cancer cells through adaptive immunity. In the metastasis link, TAMs is helpful to prepare the microenvironment suitable for tumour cells before metastasis. Targeting GS in TAM can transform TAM into classical polarization (M1), resulting in the decrease of glutamine and the increase of succinic acid in TAM. In addition, glucose flux can be increased by glycolysis to reduce glutamine metabolism, and TAM can be inhibited by inducing T cell recruitment. The ability of TAM to promote endothelial cell differentiation and cancer cell movement is impaired. Finally, the role of TAMs in promoting cancer growth and metastasis is weakened [77,78]. In a study on OSCC, it was found that c-Myc promotes the expression of GS through a positive correlation, caused more cells to be in S phase and inhibits the expression of cadherin E, promoting the metastasis of tumour cells [79]. This phenomenon has also been observed in HCC. In addition, reactive stromal cells in HCC tumour microenvironment up-regulate glutamine anabolic pathway through GS. Give stromal cells atypical metabolic flexibility and adaptive mechanism, so that they can use carbon and nitrogen from non-standard sources to synthesize glutamine under the condition of nutrient deficiency in TME. Finally promote the growth and metastasis of cancer cells [80].

## 3 Effect of glutamine on tumour metastasis through antioxidant stress

Oxidative stress refers to damage to cell DNA, proteins, and lipids caused by the excessive accumulation of intracellular ROS, which leads to cell death [81]. Iron-related death is currently one of the most relevant research topics. Iron-related death refers to the production of lipid ROS via interactions with iron ions and fatty acids in cells. Homeostasis of the intracellular environment lies in the balance between ROS and the antioxidant system, including glutathione. Glutathione is a non-protein thiol compound widely present in organisms. It is comprised of glutamic acid, cysteine, and glycine residues. Glutathione is an auxiliary factor of glutathione peroxidase 4(GPX4). Glutathione peroxidase is an enzyme that catabolizes the reduction of phospholipid hydroperoxide, which has the ability to remove membrane lipid hydroperoxide products and prevent oxidative stress. It is an important regulator of iron-related death.

Many studies have confirmed the antioxidant effect of glutathione on tumour metastasis. For example, Liu et al. confirmed that GPX4 and GSTM1 are responsible for the high consumption of GSH and reduction of ferroptosis in lung adenocarcinoma to improve the chemical resistance of tumour cells and achieve brain metastasis of primary lung cancer cells [82]. Piskounova et al. showed that a reduced GSH to oxidised glutathione (GSSG) ratio was the most important factor affecting melanoma metastasis. GSH/GSSG ratio in metastatic nodules always decreases, indicating that metastatic cells must utilise the reduced GSH against oxidative stress [83]. The cystine transporter system xCT (composed of the catalytic subunit SLC7A11 and the chaperone subunit SLC3A2) is an important transporter to communicate glutamine and glutathione. On one hand, Glutamine synthesized by GS is released from cells through this transporter; on the other hand, cystine cystine was uptaken from the extracellular environment through this transporter, and finally, cysteine was formed and used for the synthesis of glutathione. An increasing number of studies have demonstrated that xCT transporter play key roles in inhibiting oxidative reactions and maintaining cell survival and metastasis under oxidative stress conditions. They also facilitate contact between oxidative stress and cell metastasis [84]. The mechanisms and ultimate influence of high or low expression of SLC3A2 and SLC7A11 in various cancers are summarised in Table 1.

**Table 1:** Effect of glutamine transporter on tumor metastasis through oxidative stress.

Cancer	Target spot	High/Low	Signal pathway or gene	Outcome	Ref.
CRC	SLC3A2	Low	AKT/GSK- 3β	promote cell iron death and inhibit tumor growth and metastasis	[85]
LUNG		High	MEK/ERK	as a prognostic marker and induce tumor occurrence and metastasis	[86]
HCC		High	MiR-490-3p、PPM1F	promote carcinogenesis and tumor metastasis	[87]
ESCC	SLC7A11	High		inhibit cell iron death and promote tumor radio-resistance to achieve malignant progression	[88]
BRCA		Low	MiR-5096	inhibiting tumor cell colony formation, cross-pore migration, breast cancer cell invasion, and metastasis	[89]
		High	DKK1	protect metastatic cancer cells from lipid peroxidation and iron death	[90]
PDAC		Low	MiR-139-5p、P13K、Akt	inhibiting PDAC cell metastasis	[91]
		High	MCU——Keap1-Nrf2	promoting PDAC cell migration, invasion, metastasis, and metabolic stress resistance	[92]
OV		Low	MiR-382-5p	attenuate invasion and migration of ovarian cancer	[93]
CRC		Low	MELK——Akt/mTOR	exert an antitumour metastasis effect	[94]
		High	AADAC	protects metastatic colorectal cancer cells from iron death	[95]
PRAD		High	LncRNA OIP5-AS1——miR-128-3p	promotes progression and ferroptosis resistance	[96]
HCC		High	DAZAP1	promotes HCC progression and regulates ferroptosis	[97]
STAD		High	HIF-1α——lncRNA-PMAN——ELAVL1	against ferroptosis induced by Erastin and RSL3 anti-peritoneal metastasis	[98]
SKCM		Low		the metastatic potential was markedly reduced	[99]
OSCC		Low	METTL3	impaired OSCC cells proliferation, invasion, and migration	[100]
BCA		High	EMP1——PPARG	promoted cell migration and anti-ferroptotic cell death	[101]
NPC		High	ITGB3——MAPK/ERK/ATF4/Nrf2	Inhibited the ferroptosis and promoted the distant metastasis	[102]

CRC: Colon and Rectal Cancer; LUNG: Lung Cancer; HCC: Liver Cancer; ESCC: Oesophageal Cancer; BRCA: Breast Cancer; PDAC: Pancreatic Cancer; OV: Ovarian Cancer; STAD: Stomach Cancer; NPC: Nasopharyngeal Carcinoma; BCA: Bladder Cancer; OSCC: Oral Squamous Cell Carcinoma; SKCM: Skin Cutaneous Melanoma; HCC: Liver Cancer; PRAD: Prostate Cancer.

SLC3A2 (solution carrier family 3 member 2) is a type II transmembrane glycoprotein of the SLC3 family and is a heavy chain component of amino acid transporters (HATS). SLC3A2 is widely expressed and participates in the transport of most important amino acids. Increasing evidence has shown that the transport of amino acids through SLC3A2 plays an important role in the occurrence and progression of tumours.

In a study of colorectal cancer, knockdown of MIF and SLC3A2 promoted cell iron-related death and inhibited tumour growth and metastasis via the AKT/GSK-3β pathway [85]. Simultaneously, in lung cancer, SLC3A2 can induce tumour occurrence and metastasis through the MEK/ERK signalling pathway so that it can be used as a prognostic marker, and its serum content can be used to evaluate patient prognosis [86]. Its expression was also positively correlated with a low survival rate of patients with HCC [87]. In summary, SLC3A2, the partner subunit of xCT, plays an important role in protecting against oxidative stress and promoting tumour cell proliferation and metastasis.

SLC7A11 also plays an important role as a catalytic subunit of xCT in resisting oxidative stress, inhibiting cell iron-related death, and promoting tumour metastasis. In a study on ESCC, SLC7A11 expression was associated with lymph node metastasis in 127 patients with ESCC undergoing radical chemoradiotherapy. Patients with high expression of NRF2 and SLC7A11 had significantly shorter overall progression-free survival and poor response to treatment. SLC7A11 inhibits cell iron-related death and promotes tumour radioresistance to achieve malignant tumour progression [88].

In breast cancer, researchers have found that miR-5096 can induce iron-related death in breast cancer cells (Fig. 1); increase the accumulation levels of ROS, OH, lipid ROS, and iron; reduce GSH and mitochondrial membrane potential; and lead to mitochondrial atrophy and partial ridge loss, ultimately inhibiting tumour cell colony formation, cross-pore migration, invasion, and metastasis. At the same time, Yadav et al. showed through the 3′UTR luciferase test that SLC7A11 was the target of MIRI-5096, and the high expression of SLC7A11 partly restored the decrease of invasion and metastasis of breast cancer cells caused by miR-5096. The role of SLC7A11 in promoting cell survival and metastasis was demonstrated [89]. *DKK1* can be used upstream of SLC7A11 to increase the expression of SLC7A11 in breast cancer cells and protect metastatic cancer cells from lipid peroxidation and iron-related death (Fig. 1) [90].

SLC7A11 also promotes tumour cell metastasis by inhibiting iron-related death in various cancers. In pancreatic cancer, the cystine transporter SLC7A11 was identified as a druggable target downstream of the MCU-Nrf2 axis. Promoting PDAC cell migration, invasion, metastasis, and metabolic stress resistance (Fig. 1) [91]. MiR-139-5p regulated and affected the protein expression of phosphatidylinositol signalling pathway-related P13K and Akt by inhibiting SLC7A11, thereby inhibiting PDAC cell metastasis (Fig. 1) [92]. Solasonine, a steroidal alkaloid, inhibits the TFAP2A/OTUB1-SLC7A11 axis, restores SLC7A11-reduced cell iron-related death, and inhibits pancreatic cancer cell progression [103]. In ovarian cancer, lidocaine down-regulated SLC7A11 expression by increasing miR-382-5p expression (Fig. 1), to attenuate invasion and migration of ovarian cancer [93]. In colorectal cancer, xCT knockout may exert an antitumour metastatic effect by controlling the MELK-mediated activation of Akt/mTOR signalling [94]. Arylacetamide deacetylase protects metastatic colorectal cancer cells from iron-related death via slc7aA11-dependent inhibition of lipid peroxidation, thereby effectively promoting metastatic colonisation and growth in the liver [95].

In prostate cancer [96], liver cancer [97], and gastric cancer [98]; OSCC [99], melanoma [100], and bladder cancer [101]; and nasopharyngeal [102] and other cancers, SLC7A11 increases GSH levels, resists iron-related death, and promotes tumour cell metastasis respectively.

In summary, SLC3A2 and SLC7A11, which play an important role in glutathione synthesis, play a role in resisting oxidative stress, protecting cells from iron-mediated death, and promoting the survival and metastasis of tumour cells through their respective effects.

## 4 Effect of glutamine on tumour metastasis through immune evasion

Tumour cells change their metabolic characteristics and immune functions owing to environmental changes, such as external nutrition and oxygen [104]. They can inhibit the metabolism of immune T cells and affect the normal function of certain organelles by creating a unique tumour microenvironment. For example, Tumour infiltrating T cells show a continuous loss of mitochondrial function and quality due to the gradual loss of PPAR-γ coactivator 1α(PGC1α), which is a unique effect of tumour microenvironment on immune cells. Researchers have found that this phenomenon is induced by chronic Akt signal transduction in tumour-specific T cells. Reprogramming tumour-specific T cell metabolism through the forced expression of PGC1α leads to excellent intra-tumour metabolism and immune effects [105].

In addition, competition for nutrients between the tumour and immune cells in the microenvironment further limits the function of immune cells, ultimately accelerating the malignant progression of tumour cells. Blocking glucose uptake by tumour cells leads to glucose accumulation in tumour microenvironment, allowing for T-cell glycolysis and IFN-γ production, thereby playing a role in limiting the growth and metastasis of tumour cells [106]. Further research shows that, in addition to simply enhancing the metabolic activity of immune T cells, phosphoenolpyruvate, a metabolite of glycolysis, can maintain the Ca2^+^-NFAT signal and effector function mediated by T cell receptors, and the interaction between them limits the growth and distant metastasis of tumour cells [107].

Interestingly, in addition to competing for nutrients, Reinfeld et al. (2021) reported that in the tumour microenvironment, the inherent program of cells drives immune and cancer cells to obtain glucose and glutamine, respectively. However, glutamine uptake by cancer cells can inhibit glucose uptake by normal bone marrow and immune cells, creating a microenvironment suitable for tumour growth and metastasis [108]. Research on the effects of glutamine and its inhibitors on immune cells, leading to the occurrence and development of tumours, is still in its initial stages. This study expounds on the current research in this field. The mechanisms and results of glutamine inhibition by strengthening the immune response in various cancers are shown in Table 2.

**Table 2:** Effect of glutamine deficiency or blockage combined with immunotherapy on tumor cells.

Cancer	Treatment conditions	Mechanism	Outcomes	Study type	Ref.
BRCA	JHU083	1: The expression of IDO in tumor cells was inhibited, resulting in a significant decrease in the level of kynurenine. 2: The decrease of CSF3 leads to the death of recruited MDSCs and the increase of inflammatory tumor-associated Macrophages	antitumour immunity and Inhibit tumor metastasis	4T1 cells C57BL/6J BALB/CJ(CD45.1) OTI RAG1-KO BATF3-KO BALB/CJ	[109]
HCC	JHU083	1: Combined with PD1 blocker to promote immune response by increasing the ratio of CD8^+^/CD4^+^T Cell	Improve the antitumour immune responses and enhance the efficacy of immune checkpoint inhibitors	C57BL/6	[110]
LUNG	JHU083	1: Increases CD8^+^ T cell and CD4^+^ Th1 cell infiltration 2: Decreases immune suppressive cells (MDSC、regulatory T cells、pro-tumor CD4^+^ Th17 cells) 3: Upregulate the oxidative metabolism of Th1 cells and adopt a highly activated and memory-like phenotype.	Promote tumor-specific immune response mediated by adaptive T cells, improve adverse prognosis such as metastasis, and increase the effectiveness of the EGFR vaccine.	UN-SCC680 C57BL/6	[111]
MB	JHU083	1: Increases apoptosis in MYC-expressing cell lines	Increase the prognosis of the special population(Patients with MYC-expressing medulloblastoma)	D425MED MED211 Athymic nude (nu/J) C57BL/6J	[112]
PADC	L-DON	1: Adjust ECM by influencing ECM matrix remodeling enzyme and secretion factors such as IL-6 and IL-27. 2: The activation of the CD68^+^ macrophage population and the infiltration of CD8^+^T cells were promoted by increasing cytokines like IFN-Y. 3: By reducing the expression of TIM3 and CTLA-4, the sensitivity of tumor cell immunotherapy can be improved.	Deep treatment of tumours by inhibiting tumor metastasis and matrix remodeling	SU.86.86 MIAPaCa-2 Athymic nude (nu/J) C57BL/6	[113]
CTCL	dGLN L-DON AOA EGCG	1: Prevents the exhaustion and increase the number of infiltrating CD8^+^T cells 2: Increase the percentage of central memory-like cells and enhance recall response. 3: Increase the spare respiratory capacity of immune cell mitochondria.	Improve T cell immune response to cancer progression	C57BL/6	[114]

BRCA: Breast Cancer; CTCL: Cutaneous T-cell Lymphoma; PDAC: Pancreatic Cancer; HCC: Hepatocellular Carcinoma; LUNG: Lung Cancer; MB: Medulloblastoma; dGLN: deficient Glutamine; AOA: Aminooxy-acetic Acid Hemihydrochloride; EGCG: Epigallocatechin Gallate.

**Fig. 2:**
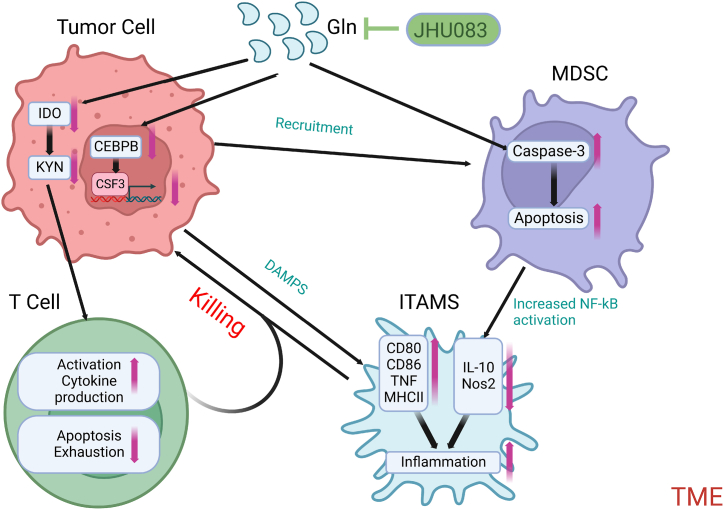
Mechanism of glutamine antagonist JHU083 enhancing antitumour immunity. Proposed models of the effect of JHU083 on the heterogeneous tumour microenvironment by blocking tumour glutamine metabolism. On the one hand, targeted glutamine metabolism inhibits IDO expression in tumour and myeloid cells, significantly decreasing kynurenine levels. This leads to the further development of immune T-cell function. On the other hand, The reduction of CEBPB leads to the reduction of CSF3 transcription, increases the death of bone-derived suppressor cells (MDSCs), and leads to an increase in inflammatory tumour-associated macrophages (TAM) by upregulating pro-inflammatory factors and decreasing anti-inflammatory factors. Immune T cells and inflammatory TAMs jointly kill tumour cells by enhancing antitumour immunity. The figures were created using Biorender.com.

In a study on intraperitoneal metastasis of ovarian cancer, researchers found that deletion of Tissue transglutaminase (TG2), an enzyme overexpressed in primary ovarian cancer, can delay intraperitoneal metastasis of ovarian cancer and reduce ascites. In addition, the deletion of TG2 can lead to an increase in CD8^+^ T cell infiltration and a decrease in the number of myeloid cells through a cytokine-induced increase in STAT1 and weakened STAT3 phosphorylation in T cells, causing metastatic cancer cells to exhibit interferon-gamma (IFN-γ)-responsive gene labelling and undergo apoptosis [115].It can inhibit tumour metastasis by combining multiple aspects [116]. In a study on triple-negative breast cancer, researchers found that by using the targeted glutamine inhibitor JHU083 (a prodrug of DON), the growth of the tumour and the production and recruitment of bone-derived suppressor cells (MDSCs) could be inhibited. On the one hand, targeted glutamine metabolism inhibits IDO expression in tumour and myeloid cells, significantly decreasing kynurenine levels. It inhibits the development of metastasis and increases the death of MDSCs and immunogenic cells by reducing CSF3, leading to an increase in inflammatory TAMs) and enhancing antitumour immunity (Fig. 2) [109].

Studies have shown that glutamine transporters play important roles in this process. In a study of HCC, IL-1β-induced SLC7A11 overexpression via the IL-1R1/ERK/SP1 pathway upregulated PD-L1 and CSF1 via the αKG/HIF1α axis. SLC7A11 promotes TAMs and MDSCs infiltration. The CSF1R inhibitor, BZL945, and anti-PD-L1 antibody blocked SLC7A11-induced HCC metastasis. Glutamine promotes tumour cell metastasis by affecting immune checkpoints and infiltration [117]. Through research on the competitive utilisation of glutamine in the microenvironment, Leone et al. found that tumour and immune cells had different outcomes in drug-induced glutamine metabolic blockade [110]. On the one hand, JHU083 can damage tumour cells' TCA cycle, purine synthesis, hexosamine pathway, and pentose phosphate pathway to different degrees.

In contrast, the blockade of glutamine metabolism increased endogenous antitumour immunity, even without concomitant immunosuppressants, which may be related to the increase in available glutamine content in the TME after blocking glutamine metabolism in tumour cells. When JHU083 was administered with the anti-PD-1 antibody, it showed a more significant antitumour effect than when administered alone [118]. Notably, almost all animals cured with single-agent JHU083 rejected the tumour upon re-attack. This suggests the establishment of immune memory, as demonstrated in RAG2^−/−^ deficient mice, which lack the V(D)J recombinase mechanism necessary to rearrange the antigen receptor gene and are therefore unable to produce mature B or T lymphocytes or adaptive immune responses [119].

In 2022, Jiang et al. pointed out that in the microenvironment, tumour cells can increase the use of glutamine by overexpressing glutamine-fructose-6-phosphate transaminase 2 to inhibit the mitochondrial fission of macrophages. However, the cytoplasmic calcium increased by mitochondrial division in macrophages can eliminate the phase transformation of the Wiskott-Aldrich syndrome protein-Wiskott-Aldrich syndrome interaction protein (WIP) complex and phosphorylate WIP through protein kinase C-θ (PKC-θ), which is a vital link for tumour cells to escape from phagocytosis [120].

Finally, several studies on distant metastasis caused by glutamine-promoted immune escape are underway. There is an increasing number of cases of glutamine inhibitors combined with various immune drugs to treat primary tumours and prevent distant metastasis [[111], [112], [113]]. Xie et al. designed a biomimetic immune metabolism nanoplatform that encapsulated a new photosensitizer and a glutamine metabolism antagonist in the cancer cell membrane to achieve specific delivery *in vivo*. This method effectively provides T cells with glutamine while inhibiting the glutamine metabolism of tumour cells, and remarkably improves the acidic, hypoxic, and low-nutritional tumour microenvironment to reprogram tumour and immune cell metabolism, induce immunogenic cell death, promote dendritic cell maturation, reduce the number of immunosuppressed cells, trigger a strong tumour-specific immune response, and regulate the tumour immunosuppressive microenvironment [121]. Furthermore, combination therapy with anti-PD-1 can produce a strong abscess effect, preventing distant metastasis of the tumour and providing long-term immune memory against tumour recurrence [114,122]. In addition, electrokinetic therapy using nanotechnology in combination with electronic currents can also be used with the glutamine antagonist 6-diazo-5-oxo-1-leucine (DON) (glutamine mimetics) to promote CD8^+^ T cell infiltration and inhibit primary or metastatic tumours [123].

## 5 Other

In addition to glutamine-metabolising enzymes, transporters, and other metabolic factors that affect immune cell function to promote metastasis, glutamine also promotes tumour cell metastasis in rare ways.

In a study on breast cancer in 2021, Torrino et al. observed that changes in the mechanical signals of the tumour microenvironment, such as enhanced tumour cell contractility, mechanical expansion of tumour blocks, and changes in the physical properties of the surrounding matrix materials, can regulate cell mechanics and affect cell metabolism to promote cancer invasion. Microtubules (MTs) have been a hot topic in recent studies of cell structure, and their biological structure determines whether they can carry large amounts of PTMs, such as acetylation, glutamylation, and glycosylation. PTMs involve the addition of a variable number of amino acid residues (e.g. glutamic acid and glycine) as secondary branches of the tubulin backbone. MTs exhibit specific cellular functions. Further studies have revealed that mechanical signals can glutamylate MTs, affect the mechanical stability of cells, and promote tumour cell metastasis, revealing the relationship between cell metabolism and MT kinetics as well as cancer invasion [124].

Glutamine has also been shown to promote tumour growth and metastasis by activating inflammatory mediators such as platelet-activating factor (PAF) [125]. Glutamine inactivates ERK, JNK, and p38 via dephosphorylation to inhibit PAF-induced angiogenic activity and decreases melanoma lung metastasis [126].

Mestre-Farrera and colleagues show that CAFs rely much more on glutamine than epithelial tumour cells. Glutamine dependence drove CAF migration toward this amino acid when cultured in low glutamine conditions: CAFs invaded a Matrigel matrix following a glutamine concentration gradient. Stimulation of glutamine-driven epithelial tumour invasion by fibroblasts required previous CAF activation. The specific mechanism includes a TGF-β/Snail1 signal axis and a polarised Akt2 distribution modulated by the Gln-dependent TRAF6 and p62 in the migrating front [127].

## 6 Conclusions

Distant metastasis, one of the cancers with the worst prognosis, significantly reduces the quality of life of patients and shortens their survival time. This has also brought great pressure and burden to modern medical systems. However, no significant progress has been made in the prevention or treatment of cancer metastases. Chemotherapy and palliative therapy do not benefit patients with most conditions. Therefore, new treatments for cancer metastasis are required [128].

With advances in the research field of nutrient metabolism in cancer metastasis, we have gradually realised the importance of metabolic substances in the occurrence and development of cancer. Compared to sugars and lipids, amino acids, the most flexible substances in the body, have more complex and diverse physiological functions. For example, glutamine plays an indispensable role in biosynthesis, antioxidant defense, chromatin modification/gene transcription, promotion of transmembrane transport of other amino acids, and regulation of cell signalling [129]. Recent studies on the relationship between glutamine-metabolising enzymes, transporters, and cancer invasiveness have fundamentally expanded our understanding of glutamine metabolism and its effects on tumour progression.

As the amino acid with the highest content in tumour cells, glutamine has various effects on tumour metabolism and malignant progression. As the most important component of glutamine metabolism, glutamine metabolic enzymes can affect tumour cell proliferation and metastasis through a variety of pathways, including regulating dry properties through ROS/Wnt/β-catenin signalling, promoting cell proliferation and metastasis through interaction with miRNAs, regulating the expression of tumour EMT-related molecules, and promoting AMPK activation and mTOR signalling inhibition to affect tumour cell metabolism and metastasis [130]. The intracellular content of glutamine and other amino acids is directly affected by glutamine transporters. Glutamine, an important component of the cellular antioxidant system, plays a significant role in oxidative stress-induced tumour metastasis. It also has indirect effects on the cell structure and inflammatory mediators.

In this study, By searching the literature about the influence of glutamine metabolism on tumour metastasis, we refer to the previous model of other nutrients affecting tumour prognosis, and take glutamine metabolism process as the starting point to elaborate the role of various key enzymes and transporters in influencing tumour metastasis in detail. Hoping to reveal the influence of glutamine on tumour metastasis through the overall process of glutamine metabolism. It puts forward new ideas and directions for studying the influence of metabolic pathways on tumour growth and prognosis. In addition, we also hope to describe the small molecular drugs targeting tumour metastasis based on glutamine metabolising enzymes and transporters.

With a continuous increase in research in this field, an increasing number of drug inhibitors of glutamine uptake and catabolism have been developed. Some of these are currently in clinical trials or have been approved by the Food and Drug Administration for use in patients with cancer or other conditions [130]. Glutamine metabolic inhibitors not only inhibit the metabolism of tumour cells but also promote the infiltration of CD8^+^ T and other immune cells and reduce the number of myeloid cells. They can also work with anti-PD-1 and other immune checkpoint inhibitors to inhibit the distant metastasis of tumour cells and enhance the effect of the microenvironment on immune injury [117].

In the future, the most effective molecular drugs for directly targeting glutamine, glutaminase, or transporter inhibitors will provide new ideas and methods for treating adverse prognoses such as tumour metastasis, which will bring substantial and effective assistance to the field of tumour cell metastasis inhibition through metabolism. In addition, they can provide patients with high drug efficacy in the clinic, thus helping to curb tumour progression [131]. In conclusion, further exploration of the effect of glutamine on tumour progression and possible strategic studies of tumour cells against glutamine restriction may reveal the intersection between metabolism and tumour progression; the deeper the understanding of reprogramming of glutamine metabolism in cancer cells, the better the development of new and efficient therapeutic intervention targets to improve tumour metastasis and drug resistance [132].

### Funding

This study was supported by grants from 10.13039/501100001809National Science Foundation of China (Grant No. 81672769) and 10.13039/501100017599Major Science and Technology Projects of Zhejiang Province (2021C03078)

## Ethics statement

Review and/or approval by an ethics committee was not needed for this study because this study does not involve ethics.

## Data availability statement

The data related to this study are not stored in a publicly available repository. No data was used for the research described in the article.

## CRediT authorship contribution statement

**Xugang Zhong:** Conceptualization, Data curation, Formal analysis, Writing – original draft, Writing – review & editing. **Zeju He:** Data curation. **Li Yin:** Project administration. **Yong Fan:** Project administration. **Yu Tong:** Project administration. **Yao Kang:** Conceptualization, Funding acquisition. **Qing Bi:** Conceptualization, Resources, Supervision.

## Declaration of competing interest

The authors declare that they have no known competing financial interests or personal relationships that could have appeared to influence the work reported in this paper.
